# Catheter Ablation Versus Medical Therapy in Atrial Fibrillation With Heart Failure With Reduced or Mildly Reduced Ejection Fraction: A GRADE‐Assessed Systematic Review and Meta‐Analysis

**DOI:** 10.1002/joa3.70464

**Published:** 2026-09-21

**Authors:** Aleena Amir Malik, Nasir Saeed, Goodluck Oparaugo, Muhammad Waqar Shahid, Waqas Kareem, Fatima Yousaf, Zain Ul Abedeen, Fatima Sajjad, Kashif Yousaf, Fizza Kazmi, Rubab Zahra, Ishfaq Ali, Sahil Kumar, Tanzeela Gul, Zartasha Rahim, Abdul Basit, Manal Sharif, Anum Noor, Habib ur Rehman, Imdad Ullah Shah, Abdullah Afridi

**Affiliations:** ^1^ Department of Medicine United Medical and Dental College Karachi Pakistan; ^2^ Department of Medicine Bacha Khan Medical College Mardan Pakistan; ^3^ Department of Public Health University of Derby Derby UK; ^4^ Department of Medicine Khyber Medical College Peshawar Pakistan; ^5^ Department of Medicine Rawalpindi Medical University Rawalpindi Pakistan; ^6^ Department of Medicine Saidu Medical College Swat Pakistan; ^7^ Department of Medicine Jalal‐Abad State University Named After B. Osmonov Medical Faculty Jalal‐Abad Kyrgyzstan; ^8^ Department of Medicine Jinnah Sindh Medical University Karachi Pakistan; ^9^ Department of Medicine Liaquat College of Medicine and Dentistry Karachi Pakistan; ^10^ Department of Medicine Ayub Medical College Abbottabad Pakistan; ^11^ Department of Medicine Liaquat National Medical College Karachi Pakistan; ^12^ Department of Medicine Saidu College of Dentistry Swat Pakistan; ^13^ Department of Medicine Quaid‐e‐Azam Medical College Bahawalpur Pakistan; ^14^ Department of Medicine Gomal Medical College D.I. Khan Pakistan

**Keywords:** atrial fibrillation, catheter ablation, heart failure, left ventricular dysfunction, meta‐analysis

## Abstract

**Background:**

Catheter ablation is an established rhythm‐control strategy for atrial fibrillation (AF), but its effectiveness in patients with heart failure with reduced or mildly reduced ejection fraction (HFrEF/HFmrEF) remains incompletely defined. We performed a GRADE‐assessed systematic review and meta‐analysis comparing catheter ablation with conventional medical therapy in this population.

**Methods:**

PubMed, Embase, and the Cochrane Library were searched from inception to February 2026. Randomized controlled trials and observational cohort studies enrolling adults with AF and left ventricular ejection fraction (LVEF) ≤ 50% were included. Primary outcomes were all‐cause mortality, cardiovascular mortality, and change in LVEF. Secondary outcomes were change in B‐type natriuretic peptide (BNP) and cardiovascular hospitalization. Random‐effects models were used, and certainty of evidence was assessed using GRADE.

**Results:**

Thirteen studies involving 131 492 participants were included. Catheter ablation significantly reduced all‐cause mortality (RR 0.61, 95% CI 0.51–0.71) and cardiovascular mortality (RR 0.46, 95% CI 0.31–0.66), improved LVEF (MD 6.47%, 95% CI 4.47–8.47), and reduced BNP levels (MD −136.85 pg/mL, 95% CI −190.46 to −83.23). No significant reduction was observed in cardiovascular hospitalization (RR 0.78, 95% CI 0.53–1.13). Mortality benefits were consistent across subgroup analyses by study design and baseline LVEF.

**Conclusions:**

Catheter ablation was associated with lower mortality and improved cardiac function compared with medical therapy in patients with AF and HFrEF/HFmrEF. Further studies specifically evaluating HFmrEF populations are warranted.

## Graphical abstract

In patients with atrial fibrillation and HFmrEF/HFrEF, catheter ablation was associated with lower mortality and improved cardiac function compared with conventional medical therapy, while cardiovascular hospitalization was not significantly reduced. The overall certainty of evidence ranged from moderate to high.

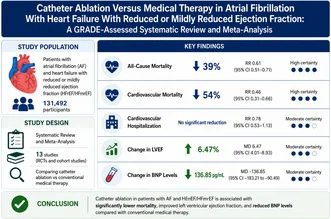

**Abbreviations**
AF: atrial fibrillation
AHRQ: Agency for Healthcare Research and Quality
BMI: body mass index
BNP: B‐type natriuretic peptide
CA: catheter ablation
CI: confidence interval
CRD: centre for reviews and dissemination
EF: ejection fraction
GRADE: grading of recommendations assessment, development, and evaluation
GRADEpro: GRADE guideline development tool
HF: heart failure
HFmrEF: heart failure with mildly reduced ejection fraction
HFrEF: heart failure with reduced ejection fraction
*I* ^2^
: higgins' I‐squared statistic
LVEF: left ventricular ejection fraction
MD: mean difference
MeSH: Medical Subject Headings
MRI: magnetic resonance imaging
NT‐proBNP: N‐terminal pro–B‐type natriuretic peptide
NYHA: New York Heart Association
PRISMA: preferred reporting items for systematic reviews and meta‐analyses
PROSPERO: International Prospective Register of Systematic Reviews
Q Test: Cochran Q test
RCT: randomized controlled trial
RevMan: review manager
ROB: risk of bias
ROB 2: risk of bias 2 tool
RR: risk ratio
ΔBNP: change in B‐type natriuretic peptide
ΔLVEF: change in left ventricular ejection fraction

## 1 Introduction

Atrial fibrillation (AF), the most common heart rhythm abnormality, is characterized by faulty electrical activity in the superior heart chambers. Similarly, heart failure (HF) stems from functional and/or structural deterioration, rendering the myocardium unable to pump enough blood, resulting in a complex clinical syndrome. Both conditions represent two intersecting, global cardiovascular epidemics that frequently coexist and share complex, self‐perpetuating pathophysiological mechanisms. These include structural myocardial remodeling, neurohormonal hyperactivation, and elevated cardiac filling pressures [1]. Patients with AF frequently develop HF, particularly in the setting of reduced or mildly reduced ejection fraction (HFrEF and HFmrEF), and vice versa [2]. Such a bidirectional relation creates a vicious cycle, exacerbating patient complaints, hemodynamic instability, stroke risk, recurrent hospitalization, and overall mortality [1, 2]. These interlaced conditions, due to severe morbidity and mortality along with the increasing global burden, remain an urgent clinical priority and warrant treatment optimization.

The standard of care in patients with concomitant AF and HFrEF or HFmrEF has long been pharmacological. These encompass drugs controlling the rate and rhythm of the heart, including beta‐blockers and digoxin, and antiarrhythmic drugs, respectively [2]. However, persistent symptoms, inability to prevent structural changes, and risk of proarrhythmogenicity and organ dysfunction, especially with long‐term use, remain valid concerns, limiting their use [3]. In contrast, catheter ablation (CA) is an emerging, disease‐modifying, invasive technique. The procedure utilizes electrical isolation to separate arrhythmogenic areas, particularly the pulmonary veins, a process known as pulmonary vein isolation. This strategy helps restore and maintain a normal sinus rhythm. Recent clinical trials show that CA is superior to conventional therapy in reducing mortality and hospitalization in the aforementioned population. However, due to a lack of statistical evidence, the consistency of these findings among variable patient demographics remains questionable [4]. Given the publication of recent trials and evolving therapeutic strategies, a novel, rigorous synthesis evaluating long‐term outcomes and optimal patient selection is required [5].

Heart failure is commonly categorized according to left ventricular ejection fraction into heart failure with reduced ejection fraction (≤ 40%), heart failure with mildly reduced ejection fraction (41%–49%), and heart failure with preserved ejection fraction (≥ 50%) [6]. Although catheter ablation has been investigated across different heart failure phenotypes, patients with reduced and mildly reduced ejection fraction share important features of ventricular systolic dysfunction and adverse ventricular remodeling and may derive greater benefit from restoration of sinus rhythm than patients with preserved ejection fraction [2, 7]. Furthermore, uncertainty remains regarding the consistency of the benefits of catheter ablation across the spectrum of non‐preserved ejection fraction, particularly among patients with heart failure with mildly reduced and reduced ejection fraction. Therefore, we conducted a systematic review and meta‐analysis comparing catheter ablation with conventional medical therapy in patients with atrial fibrillation and heart failure with reduced or mildly reduced ejection fraction (left ventricular ejection fraction ≤ 50%). In addition, the certainty of evidence for all clinically relevant outcomes was assessed using the Grading of Recommendations Assessment, Development and Evaluation (GRADE) framework. The primary objective was to evaluate the effects of catheter ablation on mortality and cardiac function, while secondary analyses examined neurohormonal and hospitalization outcomes.

## 2 Methods

### 2.1 Study Design and Protocol Registration

This systematic review and meta‐analysis adhered to the guidelines of the PRISMA (Preferred Reporting Items for Systematic Reviews and Meta‐Analyses) statement [8] and the Cochrane Handbook for Systematic Review of Interventions [9]. The stages of the review, including study identification, eligibility assessment, data collection, quality appraisal, and statistical synthesis, were performed in accordance with these methodological standards. This systematic review and meta‐analysis was registered under PROSPERO ID CRD420261359553.

### 2.2 Search Strategy and Data Sources

A comprehensive search of the literature was carried out to identify studies assessing catheter ablation in individuals with atrial fibrillation and heart failure with reduced or mildly reduced ejection fraction. The search was performed in the electronic databases PubMed, Embase, and the Cochrane Library from database inception through February 2026, without any language restrictions. The search strategy incorporated both controlled vocabulary terms, including Medical Subject Headings and Emtree terms, together with free‐text keywords. The search included combinations of terms such as “atrial fibrillation,” “AF,” “heart failure,” “congestive heart failure,” “heart failure with reduced ejection fraction,” “HFrEF,” “heart failure with mildly reduced ejection fraction,” “HFmrEF,” “reduced ejection fraction,” “left ventricular dysfunction,” “catheter ablation,” “radiofrequency ablation,” “pulmonary vein isolation,” and “cryoablation.” These search terms were combined using Boolean operators (AND, OR) to maximize the sensitivity and specificity of the search across databases. In addition to the electronic database search, the reference lists of relevant articles and previously published systematic reviews were manually screened to identify additional eligible studies.

### 2.3 Study Selection and Eligibility Criteria

All retrieved records were imported into Rayyan software for screening and management [10]. After duplicate removal, two reviewers independently evaluated titles and abstracts for potential eligibility. Studies considered potentially relevant underwent full‐text review. Disagreements regarding study inclusion were resolved through discussion between reviewers, and unresolved differences were adjudicated by a third reviewer.

Studies were considered eligible if they met the following criteria: (1) studies involving adult patients diagnosed with atrial fibrillation and heart failure with reduced or mildly reduced ejection fraction, generally defined as a left ventricular ejection fraction ≤ 50%; (2) studies with an intervention consisting of catheter ablation for atrial fibrillation; (3) a comparator consisting of conventional therapy, including pharmacological rhythm control, rate control strategies, or guideline‐directed medical therapy; (4) randomized controlled trials or observational cohort studies; and (5) studies reporting at least one clinically relevant outcome. Studies were excluded if they met any of the following criteria: (1) review articles, editorials, commentaries, conference abstracts without sufficient outcome data, case reports or case series, or in vitro experimental studies. (2) studies involving ineligible patient populations not meeting the predefined criteria; (3) duplicated datasets or republished analyses of the same cohort; (4) studies lacking an appropriate comparator group; or (5) studies not having data in extractable form.

### 2.4 Data Extraction and Outcomes

Data extraction was performed independently by two reviewers using a pre‐piloted, standardized data extraction form developed in Microsoft Excel. Any discrepancies between reviewers during the data extraction process were resolved through discussion, and when necessary, a third reviewer was consulted to achieve consensus. Baseline study characteristics and patient characteristics extracted from each included study ID (last name of the first author and year of publication), country, study duration, study design, intervention and control regimen, sample size, mean age of participants, percentage of male participants, body mass index, New York Heart Association functional class distribution, baseline left ventricular ejection fraction, and baseline comorbidities. Outcome data were extracted directly from reported results, tables, and figures in the included studies. The primary outcomes of this meta‐analysis were (1) all‐cause mortality, (2) cardiovascular mortality, and (3) change in left ventricular ejection fraction (ΔLVEF). The secondary outcomes included (1) change in B‐type natriuretic peptide levels (ΔBNP) and (2) cardiovascular hospitalization. See Table S1 for a detailed definition of outcomes.

### 2.5 Quality Assessment and Risk of Bias

The methodological quality of the included randomized controlled trials was evaluated independently by two reviewers using the Cochrane Risk of Bias 2 (ROB 2) tool [11]. This framework assesses potential bias across several domains, including the randomization process, deviations from intended interventions, missing outcome data, outcome measurement, and selective reporting. Based on these assessments, studies were categorized as having low risk of bias, some concerns, or high risk of bias. Any differences in judgment between reviewers were resolved through discussion and consensus. Application of the ROB 2 framework is consistent with approaches used in previous evidence syntheses [12].

Observational cohort studies were appraised using the Newcastle–Ottawa Scale (NOS) [13], which evaluates methodological quality according to participant selection, comparability of study groups, and outcome assessment. In accordance with Agency for Healthcare Research and Quality (AHRQ) recommendations, studies receiving 7–9 stars were considered high quality, those with 4–6 stars were regarded as moderate quality, and studies scoring 0–3 stars were classified as low quality. Assessments were conducted independently by two reviewers, with disagreements resolved through discussion or consultation with a third reviewer when required. The Newcastle–Ottawa Scale has been widely applied in systematic reviews of observational studies, including prior cardiovascular meta‐analyses [14].

### 2.6 GRADE Assessment

The Grading of Recommendations Assessment, Development, and Evaluation (GRADE) approach was applied by two independent reviewers using the GRADEpro Guideline Development Tool to assess the certainty of evidence for each outcome in this meta‐analysis [15]. The quality of evidence was categorized as high, moderate, low, or very low. Any disagreements were resolved by discussion until consensus was achieved.

### 2.7 Statistical Analysis

All statistical analyses were conducted using Review Manager (RevMan) version 5.4. For dichotomous outcomes, pooled effect estimates were calculated using risk ratios (RRs) with 95% confidence intervals (CIs). Continuous outcomes were expressed using mean differences (MDs) with corresponding 95% confidence intervals. Statistical heterogeneity across studies was evaluated using the Cochran *Q* test and *I* ^2^ statistics with *p*‐value < 0.10 and *I* ^2^ > 50% indicating significant heterogeneity [16]. A random‐effects model using the DerSimonian–Laird method was applied for all outcomes to account for between‐study variability [17]. A two‐sided *p*‐value < 0.05 was considered statistically significant. Forest plots were generated to visually present pooled effect estimates for each outcome. Sensitivity analyses were performed to evaluate the robustness of the pooled results. This included sequential exclusion of individual studies (leave‐one‐out analysis) to assess the influence of each study on the overall effect estimate. Additional subgroup analyses were conducted according to study design (randomized controlled trials vs. observational cohort studies) to explore the potential influence of study design on pooled treatment effects and to investigate sources of clinical heterogeneity. Differences between subgroups were assessed using the test for subgroup differences. An additional subgroup analysis for the primary outcomes was performed according to the mean baseline left ventricular ejection fraction (LVEF) of the included study populations (< 40% vs. ≥ 40%) to explore whether baseline systolic function influenced treatment effects. Publication bias was assessed for outcomes reported in at least 10 studies using visual inspection of funnel plots and Egger's regression test for funnel plot asymmetry. Consistent with conventional recommendations, a *p* value < 0.10 was considered indicative of potential small‐study effects. Post hoc sensitivity analyses were conducted when substantial heterogeneity was identified to evaluate the influence of individual studies on the pooled effect estimates.

## 3 Results

### 3.1 Search Results

**FIGURE 1:**
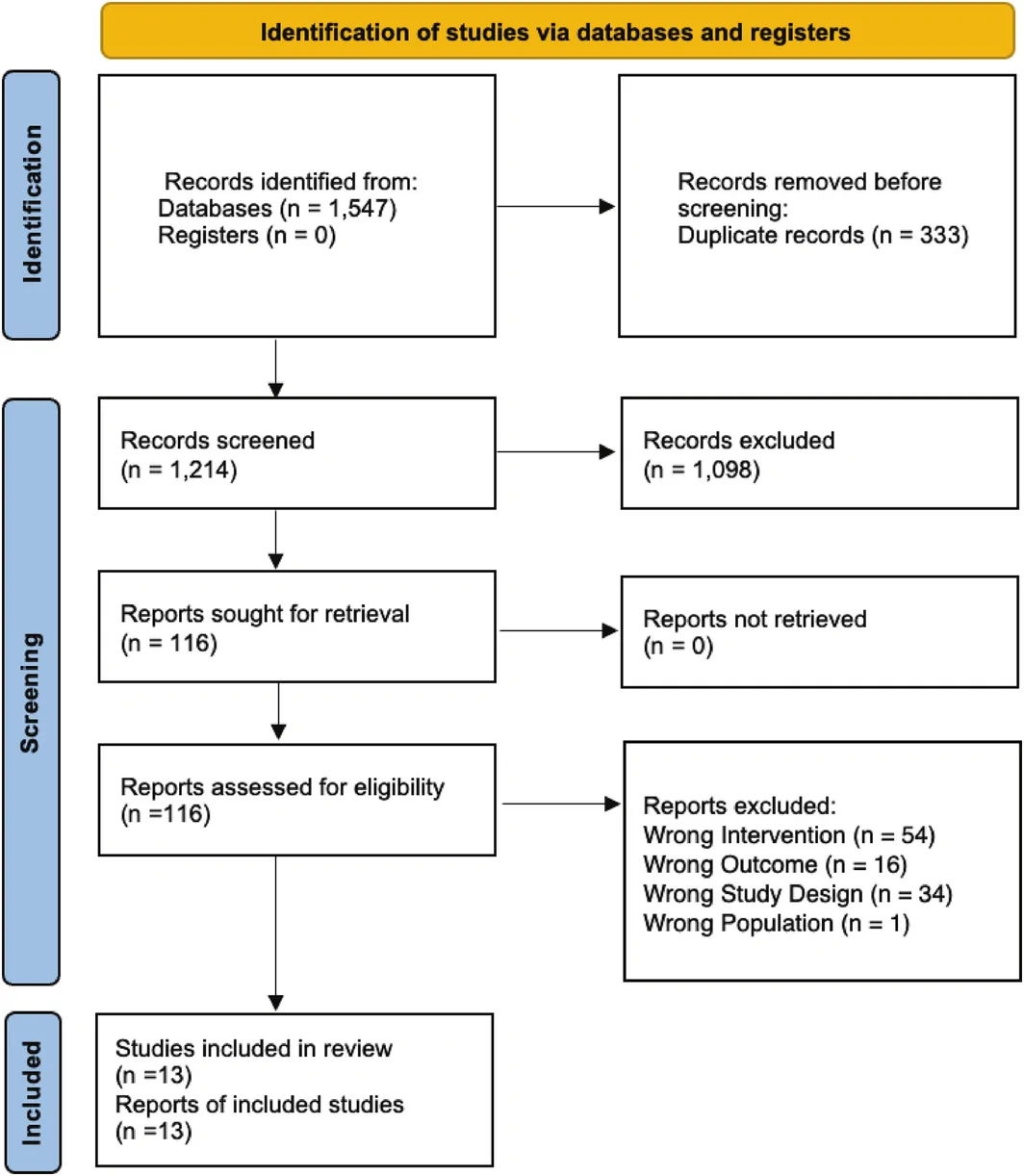
PRISMA flowchart.

The literature search identified 1547 records from electronic databases (PubMed, Embase, and the Cochrane Central Register of Controlled Trials [CENTRAL]). After removal of 333 duplicate records, 1214 records remained for title and abstract screening. Of these, 1098 records were excluded, and 116 full‐text articles were assessed for eligibility. Following full‐text review, 105 articles were excluded due to an inappropriate intervention (*n* = 54), an inappropriate outcome (*n* = 16), an inappropriate study design (*n* = 34), and inappropriate population (*n* = 1). Ultimately, 13 studies met the eligibility criteria and were included in the qualitative and quantitative synthesis (Figure 1) [7, 18, 19, 20, 21, 22, 23, 24, 25, 26, 27, 28, 29].

### 3.2 Study and Baseline Characteristics

A total of 13 studies involving 131 492 participants (65 739 in the catheter ablation group and 65 753 in the conventional therapy group) were included in this meta‐analysis. Of these, 10 were randomized controlled trials, including one long‐term follow‐up of a randomized controlled trial, and three were retrospective observational cohort studies. The studies were conducted across North America, Europe, Asia, and Australia, with recruitment periods spanning 2005 to 2024. Catheter ablation, primarily involving pulmonary vein isolation with or without additional lesion sets, was compared with conventional medical therapy, including rate‐control or rhythm‐control strategies, antiarrhythmic drug therapy, or guideline‐directed heart failure management. Follow‐up duration ranged from 6 months to 8 years.

Baseline demographic and clinical characteristics were generally comparable between the CA and CT groups across the included studies. The mean age of participants ranged from 55 to 74 years, and most study populations were predominantly male (60%–96%). Mean body mass index, where reported, ranged from 24.4 to 30.7 kg/m^2^, while baseline left ventricular ejection fraction ranged from 16.1% to 45.5%, reflecting patients with predominantly reduced systolic function. Most participants had NYHA Class II or III heart failure at enrollment. Common comorbidities included hypertension (31%–65%), diabetes mellitus (11%–35%), and coronary artery disease (13%–65%). Overall, baseline characteristics were well balanced between the intervention and control groups within individual studies. Study and baseline characteristics of the included populations are presented in Tables 1 and 2.

**TABLE 1:** Study characteristics.

Author, year	Recruitment period	Country	Type of study	Intervention	Control	Sample size		Follow‐up
						CA	CT	
Biase 2016	Aug 2008–Dec 2014	USA, Italy, France, Czech Republic	RCT	Catheter ablation	Amiodarone (AMIO) therapy	102	101	24 months
Hunter 2014	Mar 2005–Oct 2011	United Kingdom	RCT	Catheter ablation (pulmonary vein isolation with additional lesion sets as required)	Medical therapy (rate control ± standard heart failure treatment)	26	24	12 months
Jones 2013	Apr 2009–Jun 2012	United Kingdom	RCT	Catheter ablation: pulmonary vein isolation, linear lesions (roof and mitral isthmus), ablation of complex fractionated electrograms	Rate control with beta‐blockers and/or digoxin targeted to mean HR ≤ 80 bpm at rest and ≤ 110 bpm after 6‐min walk	26	26	12 months
Kuck 2019	Jan 2008–Jun 2016	Germany, Hungary, Spain	RCT	Catheter ablation (mandatory circumferential PV isolation; additional linear or complex fractionated electrogram ablation allowed at investigator discretion) plus optimized medical therapy	Best medical therapy (BMT) alone according to ACC/AHA/ESC 2006 guidelines for AF, including rate or rhythm control and device therapy as indicated	68	72	12 months
Lee 2022	Jun 2005–Feb 2020	Taiwan	Retrospective, single‐center observational Cohort study	Catheter ablation	Medical Therapy	72	72	19 months
Long 2019	May 2006–Apr 2013	China	Single‐center, retrospective cohort	Catheter ablation	Medical therapy	120	150	12 months
Macdonald 2010	Jan 2007–Jul 2009	United Kingdom	RCT	Radiofrequency ablation (RFA) + continued medical therapy	Medical therapy alone (optimal HF treatment ± digoxin for rate control)	22	19	6 months
Marrouche 2018	Jan 2008–Jan 2016	Europe, Australia, United States	RCT	Catheter ablation (pulmonary vein isolation ± additional lesions)	Medical therapy (rate control or rhythm control according to guidelines)	179	184	38 months
Nor 2025	Jan 2014–Dec 2024	United States	Retrospective Cohort	Catheter ablation (pulmonary vein isolation or other AF ablation procedures)	Conventional medical therapy (rate or rhythm control medications without ablation)	64 743	64 743	36 months
Parkash 2022	Dec 2011–Jan 2018	Brazil, Canada, Sweden, Taiwan	RCT	Ablation‐based rhythm control (catheter ablation ± antiarrhythmic drugs)	Rate control (medical therapy ± AV node ablation with pacing if needed)	214	197	37 months
Prabhu 2018	Nov 2013–Oct 2016	Australia	RCT	Catheter ablation (pulmonary vein isolation with additional lesions; rhythm control strategy)	Medical rate control (pharmacological therapy to control ventricular rate)	18	18	6 months
Sohns 2023	Nov 2020–May 2022	Germany	RCT	Catheter ablation + guideline‐directed medical therapy	Medical therapy alone (rate/rhythm control per guidelines)	97	97	18 months
Zakeri 2022	CAMTAF: Jun 2005–Jul 2011; ARC‐HF: Apr 2009–Jun 2012	United Kingdom	Long‐term follow‐up of a RCT	Catheter ablation (early or delayed; pulmonary vein isolation ± additional lesions)	Pharmacological rate control (β‐blockers, digoxin ± guideline‐directed HF therapy)	52	50	8 years

**TABLE 2:** Patient characteristics.

	Age (yrs) mean (SD)		Male (%)		BMI (kg/m^2^) mean (SD)		NYHA class (%)		LVEF mean (SD)		Hypertension (%)		Diabetes (%)		Coronary artery disease (%)	
	CA	CT	CA	CT	CA	CT	CA	CT	CA	CT	CA	CT	CA	CT	CA	CT
Biase 2016	62 (10)	60 (11)	75	73	30 (8)	29 (4)	NR	NR	29 (5)	30 (8)	45	48	22	24	62	65
Hunter 2014	55 (12)	60 (10)	96	96	NR	NR	II: 42 III: 58	II: 50 III: 50	31.8 (7.7)	33.7 (12.1)	31	33	NR	NR	NR	NR
Jones 2013	64 (10)	62 (9)	81	92	NR	NR	II: 54 III: 46	II: 50 III: 50	22 (8)	25 (7)	NR	NR	NR	NR	NR	NR
Kuck 2019	65 (8)	65 (8)	88	92	29.4 (5.0)	28.4 (4.5)	II: 41 III: 59	II: 38 III: 62	27.8 (9.5)	24.8 (8.8)	NR	NR	35	31	NR	NR
Lee 2022	59 (7.5)	62 (6.8)	79	67	25.16 (3.9)	24.37 (4.2)	NR	NR	45.5 (2.7)	45 (2.7)	61	49	11	15	15	21
Long 2019	61 (9.1)	63 (8.5)	60	60	NR	NR	NR	NR	41.9 (4.6)	40.8 (4.8)	60	63	16	17	13	17
Macdonald 2010	62 (6.7)	64 (8.3)	77	79	30 (5.6)	30 (5.7)	II: 9 III: 91	II: 11 III: 89	16.1 (7.1)	19.6 (5.5)	64	58	32	21	NR	NR
Marrouche 2018	64 (11.2)	65 (13)	87	84	29.0 (4.7)	29.1 (4.7)	I: 11 II: 58 III: 29 IV: 2	I: 11 II: 61 III: 27 IV: 1	31.8 (9.71)	31.9 (7.47)	NR	NR	NR	NR	NR	NR
Nor 2025	73 (11.4)	74 (11.5)	64	64	NR	NR	NR	NR	NR	NR	60	60	21	21	NR	NR
Parkash 2022	66 (8.6)	68 (8.0)	73	75	30.1 (6.5)	30.7 (6.7)	II: 67 III: 33	II: 67 III: 33	40.9 (15)	40.3 (14.7)	65	67	29	33	NR	NR
Prabhu 2018	59 (13)	63 (7.1)	NR	NR	31 (8.7)	30 (2.7)	NR	NR	33 (8)	36 (8.2)	44	22	17	28	NR	NR
Sohns 2023	62 (12)	65 (10)	88	74	28 (4)	28 (5)	II: 34 III: 54 IV: 12	II: 29 III: 56 IV: 15	29 (6)	25 (6)	NR	NR	26	32	NR	NR
Zakeri 2022	60 (14.8)	63 (8.9)	89	94	NR	NR	II: 48 III: 52	II: 50 III: 50	28.9 (7.62)	33 (14.6)	31	34	NR	NR	33	42

Abbreviations: BMI, body mass index; CA, catheter ablation; CT, conventional therapy; LVEF, left ventricular ejection fraction; NR, not reported; NYHA, New York Heart Association.

### 3.3 Risk of Bias Assessment

The methodological quality of RCTs was assessed using the ROB 2.0 tool. Of the included RCTs, 7 studies were classified as low risk of bias while 4 studies demonstrated some concerns. Some concerns arose mainly in the randomization process or deviations from intended interventions domain. Importantly, no study was classified as having a high risk of bias, reflecting strong methodological quality (Figures S1 and S2). The methodological quality of the three included cohort studies was assessed using the Newcastle–Ottawa Scale (NOS). Lee et al. achieved 9 stars, indicating high methodological quality and a low risk of bias. Long et al. and Nor et al. each received 7 stars, reflecting good methodological quality. Overall, all included cohort studies were considered to be of moderate‐to‐high quality, with NOS scores ranging from 7 to 9 stars (Table S2).

### 3.4 GRADE Assessment

The certainty of evidence for the primary and secondary outcomes was evaluated using the GRADE framework. All outcomes were supported by moderate‐ to high‐certainty evidence. All‐cause mortality and cardiovascular mortality were rated as high‐certainty evidence across all GRADE domains. Change in left ventricular ejection fraction (ΔLVEF), change in B‐type natriuretic peptide (ΔBNP) levels, and cardiovascular hospitalization were rated as moderate‐certainty evidence. ΔLVEF and cardiovascular hospitalization were downgraded due to substantial heterogeneity across studies, whereas ΔBNP was downgraded because of imprecision resulting from the small sample size. Detailed GRADE assessments are presented in Table S3.

### 3.5 Meta‐Analysis of Primary Outcomes

#### 3.5.1 All‐Cause Mortality

**FIGURE 2:**
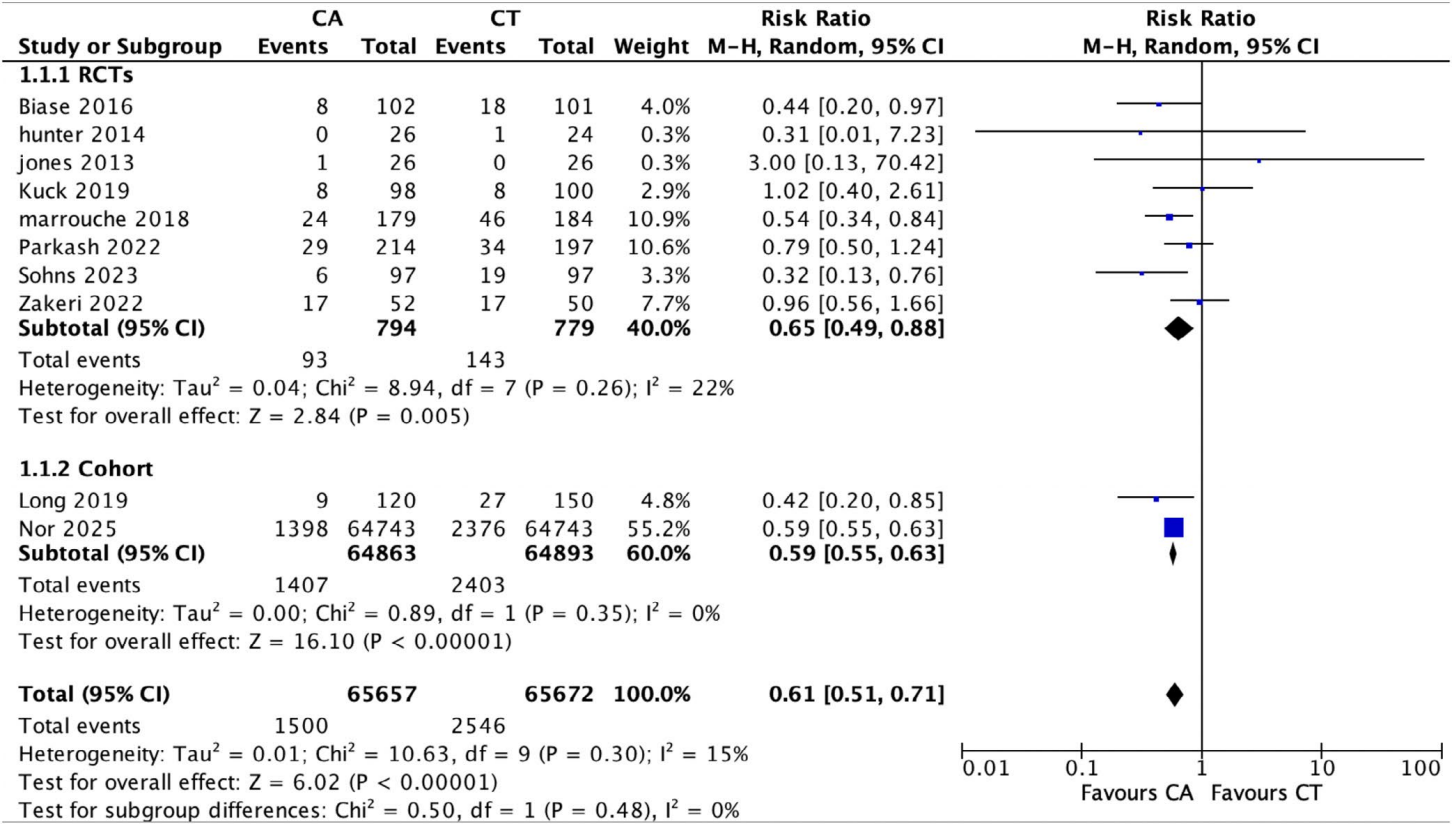
Forest plot of all cause mortality.

All‐cause mortality was reported in 10 studies including 131 329 participants (catheter ablation: *n* = 65 657; medical therapy: *n* = 65 672). Pooled analysis demonstrated that catheter ablation was associated with a significant reduction in all‐cause mortality compared with medical therapy (RR 0.61, 95% CI 0.51–0.71; *p* < 0.00001) (Figure 2). Low heterogeneity was observed across studies (*I* ^2^ = 15%). Subgroup analysis according to study design demonstrated a significant reduction in all‐cause mortality with catheter ablation in both randomized controlled trials (RR 0.65, 95% CI 0.49–0.88; *I* ^2^ = 22%) and observational cohort studies (RR 0.59, 95% CI 0.55–0.63; *I* ^2^ = 0%), with no significant subgroup difference (*p* for subgroup interaction = 0.48). Subgroup analysis according to mean baseline left ventricular ejection fraction (LVEF) demonstrated a significant reduction in all‐cause mortality among studies with a mean baseline LVEF < 40% (RR 0.60, 95% CI 0.50–0.72; *I* ^2^ = 15%), whereas no significant reduction was observed among studies with a mean baseline LVEF ≥ 40% (RR 0.61, 95% CI 0.33–1.12; *I* ^2^ = 54%). There was no significant subgroup difference between baseline LVEF categories (*p* for subgroup interaction = 0.96) (Figure S3). Assessment of publication bias using the funnel plot showed no obvious asymmetry. Furthermore, Egger's regression test was not statistically significant (*p* = 0.735), suggesting no evidence of publication bias for the all‐cause mortality outcome (Figure S4).

#### 3.5.2 Cardiovascular Mortality

**FIGURE 3:**
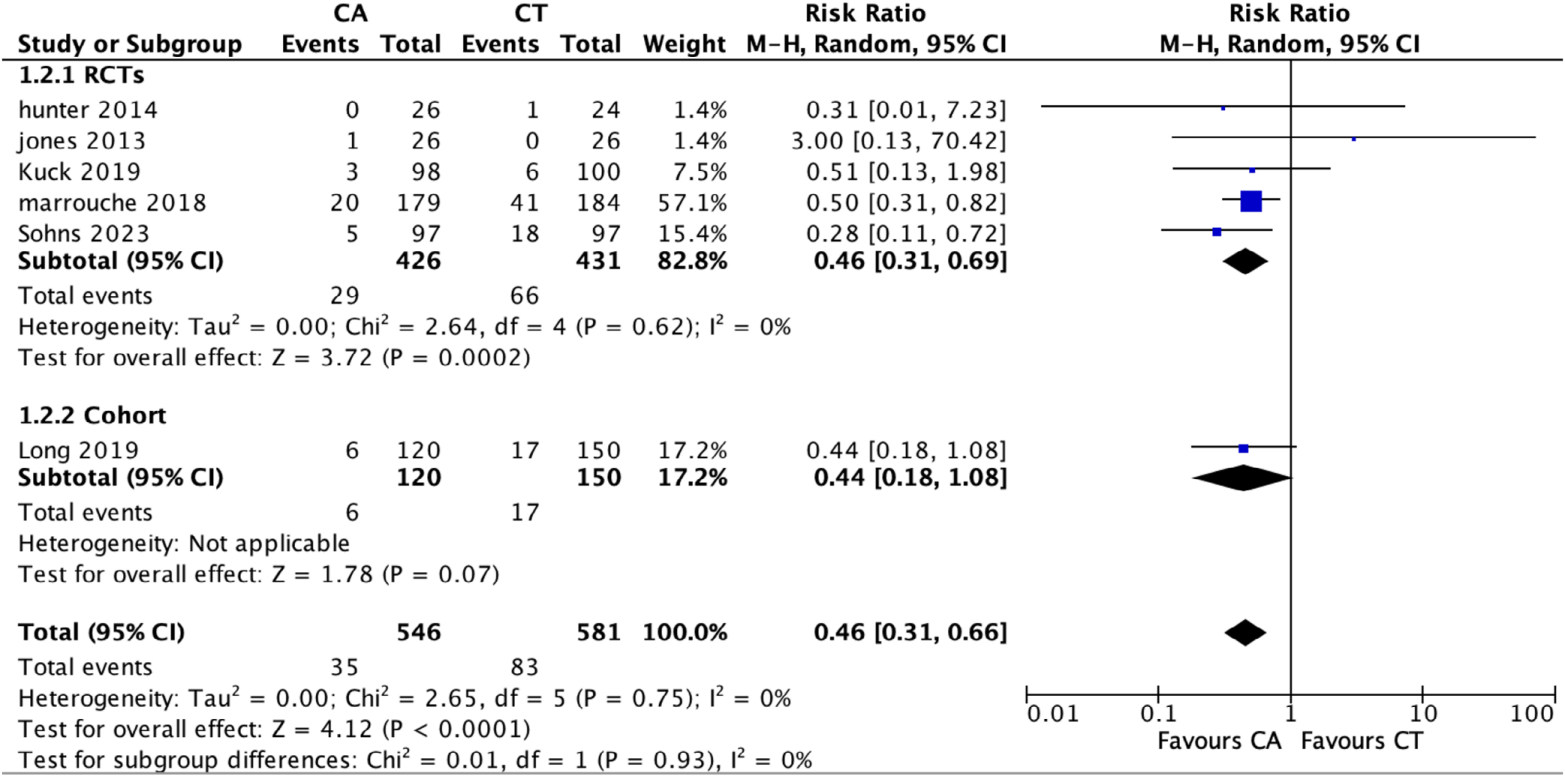
Forest plot of cardiovascular mortality.

Cardiovascular mortality was reported across six studies including 1127 participants (catheter ablation: *n* = 546; medical therapy: *n* = 581). Catheter ablation was associated with a significant reduction in cardiovascular mortality compared with medical therapy (RR 0.46, 95% CI 0.31–0.66; *p* < 0.0001) (Figure 3). No statistical heterogeneity was observed (*I* ^2^ = 0%). Subgroup analyses according to study design and mean baseline LVEF demonstrated no significant subgroup differences (*p* for subgroup interaction = 0.93 for both analyses) (Figure S5).

#### 3.5.3 Change in Left Ventricular Ejection Fraction (ΔLVEF)

**FIGURE 4:**
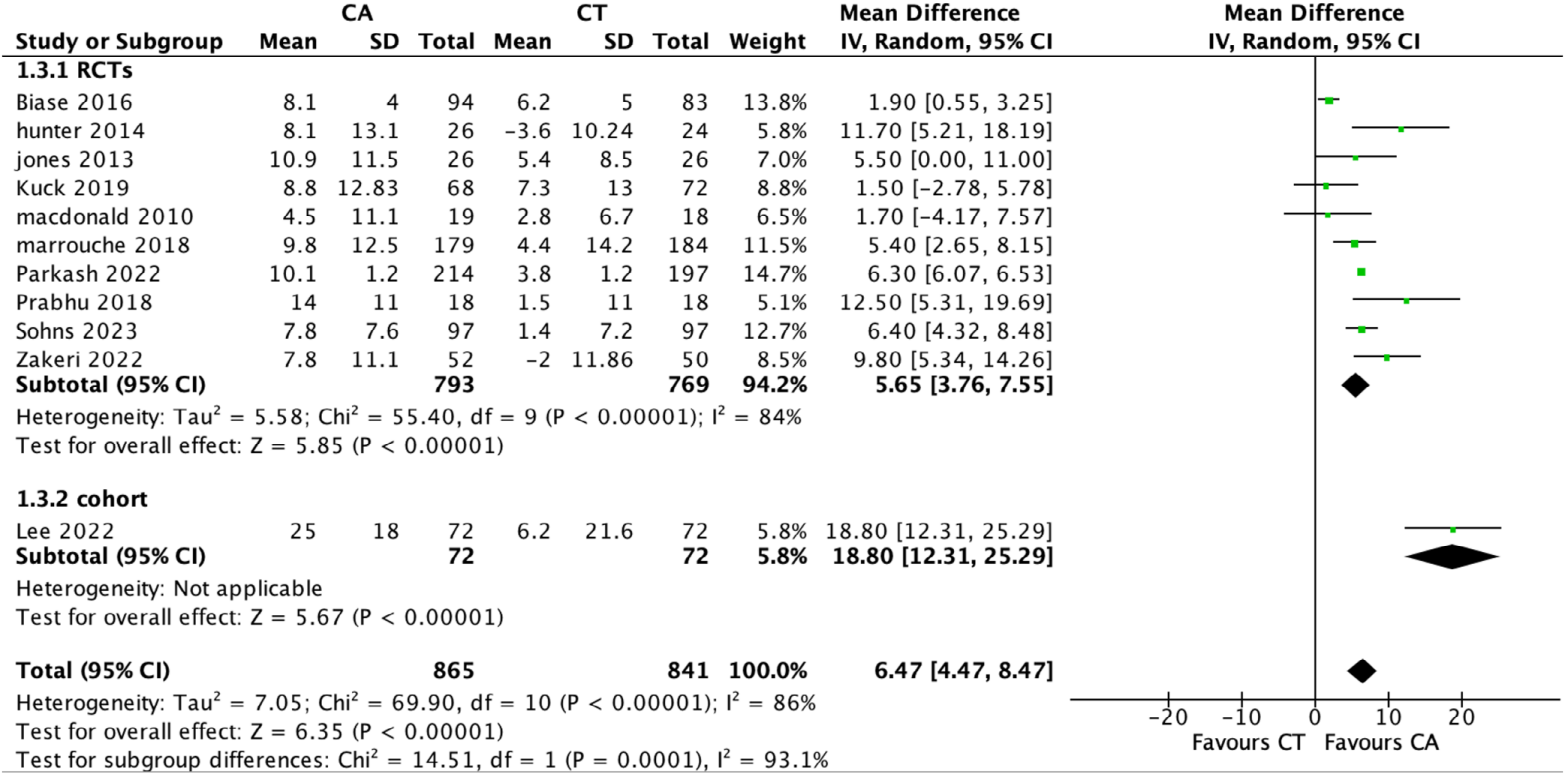
Forest plot of change in left ventricular ejection fraction.

A total of 11 studies including 1706 participants (865 in the ablation group and 841 in the control group) reported change in LVEF. Catheter ablation significantly improved LVEF (MD +6.47%, 95% CI 4.47–8.47; *p* < 0.00001) (Figure 4). However, substantial heterogeneity was present (*I* ^2^ = 86%). Heterogeneity persisted despite doing leave‐one‐out analysis. This likely suggests that the variability reflects differences in study characteristics rather than the influence of any single study. Assessment of publication bias revealed no marked funnel plot asymmetry, and Egger's regression test was not significant (*p* = 0.927). Nevertheless, interpretation should be made with caution given the substantial between‐study heterogeneity observed for this outcome (*I* ^2^ = 86%) (Figure S6). Subgroup analysis according to study design demonstrated a significant subgroup difference (*p* for subgroup interaction = 0.0001), whereas no significant subgroup difference was observed according to mean baseline LVEF (*p* for subgroup interaction = 0.31) (Figure S7).

### 3.6 Meta‐Analysis of Secondary Outcomes

#### 3.6.1 Change in B‐Type Natriuretic Peptide (BNP)

**FIGURE 5:**
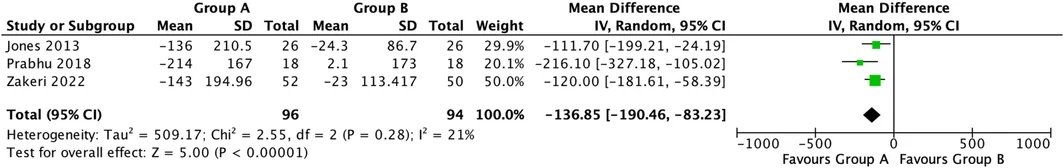
Forest plot of change in B‐type natriuretic peptide.

Change in B‐type natriuretic peptide (BNP) concentration (pg/mL) was assessed in 3 studies, encompassing 190 patients (96 in the ablation group vs. 94 in the comparator group). Catheter ablation was associated with a significant reduction in BNP levels (MD −136.85 pg/mL, 95% CI −190.46 to −83.23; *p* < 0.00001) (Figure 5), with low heterogeneity (*I* ^2^ = 21%), indicating consistent effects across studies. To ensure methodological consistency, studies reporting NT‐proBNP were excluded from this analysis.

#### 3.6.2 Cardiovascular Hospitalization

**FIGURE 6:**
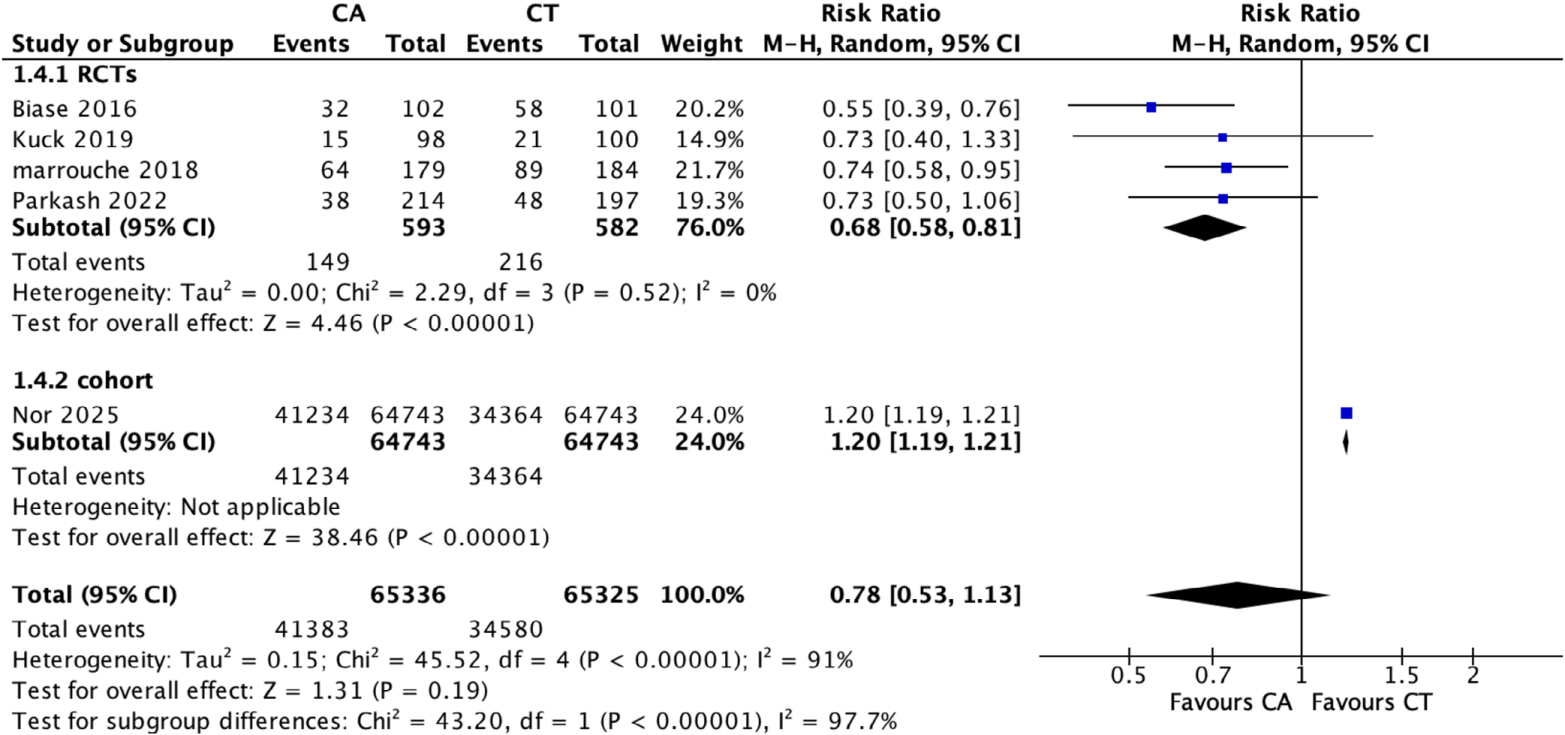
Forest plot of cardiovascular hospitalization. CA, catheter ablation; CT, conventional therapy.

Five studies including 130 661 participants reported cardiovascular hospitalization. In the overall analysis, there was no significant difference between catheter ablation and medical therapy (RR 0.78, 95% CI 0.53–1.13; *p* = 0.19), with considerable heterogeneity (*I* ^2^ = 91%) (Figure 6). Subgroup analysis according to study design demonstrated a significant subgroup difference (*p* for subgroup interaction < 0.00001). A post hoc sensitivity analysis excluding the observational cohort by Nor et al. demonstrated a significant reduction in cardiovascular hospitalization with catheter ablation (RR 0.68, 95% CI 0.58–0.81; *p* < 0.00001), with no observed heterogeneity (*I* ^2^ = 0%) (Figure S8).

## 4 Discussion

This study examines the comparative impact of catheter ablation versus conventional medical therapy in patients with atrial fibrillation and heart failure with mildly reduced or reduced ejection fraction (LVEF ≤ 50%), with a focus on mortality, ventricular function, neurohormonal response, and cardiovascular hospitalization. Many previous meta‐analyses have largely been restricted to patients with severely reduced ejection fraction or randomized trial data alone, thereby restricting generalizability across the broader spectrum of ventricular dysfunction [30, 31, 32, 33, 34]. In contrast, other studies that incorporated mixed heart failure populations showed heterogeneity in their outcomes [2, 35, 36]. Furthermore, several randomized and observational studies, including long‐term follow‐up data, have been published since these earlier evidence syntheses. By specifically focusing on patients with LVEF ≤ 50% and including both randomized and observational evidence, this analysis provides a comprehensive evaluation of catheter ablation across a broader yet clinically relevant spectrum of ventricular dysfunction. The expanded evidence base also enabled predefined subgroup analyses according to study design and baseline LVEF, providing additional insight into the consistency of treatment effects across different methodological designs and degrees of ventricular dysfunction. This large pooled analysis consisting of 131 492 patients across 13 studies (10 randomized controlled trials, including one long‐term follow‐up of a randomized controlled trial, and three observational cohort studies) demonstrated that catheter ablation was associated with significant reductions in both all‐cause and cardiovascular mortality, alongside improvements in left ventricular ejection fraction and BNP levels. The subgroup analyses demonstrated that the mortality benefit remained consistent irrespective of study design or baseline LVEF, thereby strengthening the robustness and generalizability of these findings. While the primary analysis did not demonstrate a statistically significant reduction in cardiovascular hospitalization, additional subgroup analyses according to study design suggested that methodological differences between randomized controlled trials and observational studies contributed to the observed heterogeneity. Furthermore, a post hoc sensitivity analysis excluding the large observational cohort demonstrated a significant reduction in cardiovascular hospitalization with complete resolution of heterogeneity; however, this exploratory finding should be interpreted cautiously. These findings suggest that catheter ablation may influence disease progression beyond symptom control in patients with atrial fibrillation and impaired ventricular function.

The observed benefits are biologically plausible and supported by well‐established pathophysiological mechanisms. Atrial fibrillation accelerates and exacerbates ventricular dysfunction via mechanisms such as loss of atrioventricular synchrony, irregular ventricular filling, and tachycardia‐mediated cardiomyopathy. Concomitantly, heart failure induces atrial remodeling, which promotes perpetuation of arrhythmia. Catheter ablation restores sinus rhythm more effectively than pharmacological therapy, thereby improving ventricular filling dynamics, reducing myocardial oxygen demand, and attenuating neurohormonal activation. These effects have been demonstrated in mechanistic and clinical studies evaluating rhythm control strategies, which show improvements in ventricular function, myocardial energetics, and reverse remodeling following successful ablation [37, 38]. These mechanisms are further reflected in the observed improvements in LVEF and reductions in BNP in our analysis.

One important consideration is the imbalance in sample size between randomized and observational studies. The observational cohort by Nor et al. contributed a substantial proportion of participants, particularly to the analyses of all‐cause mortality and cardiovascular hospitalization, and therefore carried considerable statistical weight in the pooled estimates. To address this concern, subgroup analyses according to study design were performed. The mortality benefits remained consistent across randomized controlled trials and observational studies, with no significant subgroup differences. However, significant subgroup differences were observed for left ventricular ejection fraction and cardiovascular hospitalization, indicating that study design contributed to the observed heterogeneity. Accordingly, these findings should be interpreted in light of the inherent risk of residual confounding associated with non‐randomized studies. Although this review included patients with both HFrEF and HFmrEF (LVEF ≤ 50%), most included studies predominantly enrolled patients with HFrEF. Subgroup analyses according to baseline LVEF (< 40% vs. ≥ 40%) showed no significant differences in mortality outcomes; however, the limited representation of HFmrEF populations warrants cautious interpretation and confirmation in dedicated HFmrEF studies.

Another important consideration is the clinical heterogeneity among the included studies. Although catheter ablation was evaluated as a single intervention, important differences existed in ablation strategies (pulmonary vein isolation alone vs. additional lesion sets and substrate modification), ablation technologies and procedural eras, atrial fibrillation subtype, baseline ventricular function, follow‐up duration, and background heart failure therapy. These variations reflect the evolution of catheter ablation techniques and heart failure management over time and may have influenced treatment effects. However, detailed subgroup analyses according to these characteristics were not feasible because these variables were inconsistently reported across the included studies. Therefore, the pooled estimates should be interpreted in the context of this underlying clinical heterogeneity.

Importantly, our observed mortality benefit appeared similar to that of previous meta‐analyses of randomized controlled trials. Earlier RCT‐based meta‐analyses showed significant relative reductions in all‐cause mortality with catheter ablation with RRs of around 40%–50% [30, 31, 32]. More recent analyses have confirmed these findings, reporting sustained reductions in mortality and cardiovascular events with low to moderate heterogeneity [33, 34, 37]. The comprehensive meta‐analysis by Zhang et al. [39] which is the largest meta‐analysis in this field to date (777 668 patients) reported almost identical results for all‐cause mortality (RR 0.57) to our analysis (RR 0.61) [39]. Together, these previous meta‐analyses suggest a consistent survival benefit with catheter ablation that appears to be durable. Consistent with previous studies, pooled estimates of improvement in left ventricular ejection fraction ranged from around 4% to 7% in prior meta‐analyses [30, 33, 37]. The stronger effect (MD +6.47%) in this analysis was likely larger due to the inclusion of studies that recruited patients with LVEF up to 50%, who are more likely to exhibit reverse remodeling due to greater myocardial reserve. This is supported by prior subgroup analyses by Cui et al. [40], demonstrating greater LVEF improvement in patients with higher baseline ejection fraction [40]. Additionally, there was high heterogeneity for this outcome, which did not resolve despite leave‐one‐out sensitivity analysis, suggesting that the observed variability was not driven by any single study. This finding was consistent with one of the previous studies [37]. The observed heterogeneity likely reflects both clinical and methodological differences across studies, including variations in baseline ventricular function, atrial fibrillation subtype and duration, follow‐up duration, and imaging modalities used for LVEF assessment. Although all studies reported LVEF as a percentage, permitting pooling using mean differences, differences in imaging techniques, particularly echocardiography versus cardiac magnetic resonance imaging, may have contributed to measurement variability and the observed heterogeneity.

Significant reduction in BNP levels further supports the physiological benefits of catheter ablation. BNP is a sensitive marker of increased ventricular wall stress and of activation of the neurohormonal system, and therefore, decreased BNP levels indicate improvement in cardiac function. The BNP reduction of −136.85 pg/mL in this analysis is methodologically distinctive due to the deliberate exclusion of NT‐proBNP studies, ensuring biomarker comparability across the three contributing studies. Because all included studies reported BNP in the same unit (pg/mL), pooling using mean differences was considered appropriate. Nevertheless, residual methodological variability related to assay platforms, laboratory methods, and baseline patient characteristics may have influenced the pooled estimates despite restricting the analysis to BNP‐only studies. These findings are directionally consistent with Zhang et al. [39] who reported a pooled BNP reduction of −120.54 pg/mL (95% CI −160.85 to −80.22; *I* ^2^ = 0%) across five studies incorporating both BNP and NT‐proBNP after subgroup stratification [39].

Cardiovascular hospitalization reported high heterogeneity in our analysis. While prior meta‐analyses have generally reported a reduction in heart failure hospitalizations following catheter ablation [30, 33, 34], no significant reduction was observed for cardiovascular hospitalization in the primary analysis. This variable outcome likely results from several limitations, including the inclusion of observational studies versus randomized trials, and the differences between time‐to‐event versus dichotomous outcome reporting. However, exclusion of a single methodologically divergent study eliminated heterogeneity and revealed a significant reduction in hospitalization risk, aligning closely with prior randomized evidence. These findings suggest that methodological differences, together with underlying clinical heterogeneity among the included studies, may have contributed to the observed variability in hospitalization outcomes. It is important to note that LVEF and BNP were not the primary efficacy endpoints in most of the included studies but were generally reported as secondary or exploratory outcomes to assess cardiac function and reverse remodeling. Consequently, these outcomes were not consistently prespecified, uniformly powered, or assessed using identical protocols across studies. Nevertheless, because they represent clinically relevant markers of ventricular function and neurohormonal activity and were reported using consistent measurement scales, quantitative synthesis was considered appropriate. However, these findings should be interpreted as supportive evidence of physiological benefit rather than definitive estimates of treatment effect. While statistical heterogeneity, as reflected by the I^2^ statistic, was observed for several outcomes, clinical heterogeneity also existed across the included studies owing to differences in patient populations, baseline LVEF, atrial fibrillation subtype, ablation techniques, comparator strategies, follow‐up duration, and outcome definitions. These sources of clinical variation should be considered alongside statistical heterogeneity when interpreting the pooled estimates.

These findings align with contemporary meta‐analyses demonstrating consistent reductions in mortality and heart failure events with catheter ablation across randomized populations [33, 34]. However, the included studies were conducted over more than a decade, during which catheter ablation techniques, mapping systems, energy delivery strategies, and operator experience evolved considerably. Consequently, procedural efficacy and safety may differ between earlier and more contemporary studies, which could influence the observed treatment effects. By incorporating recently published studies and performing subgroup analyses according to study design and baseline LVEF, the present analysis further strengthens the evidence supporting these benefits across a broader spectrum of patients with heart failure. The consistency of these effects supports a durable benefit of rhythm control beyond short‐term symptomatic improvement. The findings of this study have important clinical implications. In addition to improving symptoms and rhythm control, catheter ablation was associated with reductions in mortality and improvements in ventricular function and neurohormonal status. These findings support consideration of catheter ablation as an important therapeutic strategy in appropriately selected patients with AF and HFmrEF/HFrEF, particularly when maintenance of sinus rhythm is a major treatment objective. These observations suggest that the benefits of catheter ablation may extend beyond symptom relief and may contribute to modification of the underlying disease process.

## 5 Strength and Limitation

This meta‐analysis has several strengths. First, it includes a large and geographically diverse population of 131 492 patients from studies conducted across North America, Europe, Asia, and Australia, enhancing the generalizability of the findings. Second, by integrating randomized controlled trials and high‐quality observational cohort studies, this review provides a comprehensive assessment of both efficacy and real‐world effectiveness. Third, subgroup analyses according to study design (randomized controlled trials vs. observational cohort studies) and baseline left ventricular ejection fraction (< 40% vs. ≥ 40%) provided additional insight into the consistency of treatment effects across different methodological designs and patient populations. Fourth, the methodological quality of the included studies was high, with no study judged to have a high risk of bias, and all outcomes were evaluated using the GRADE framework, demonstrating moderate‐ to high‐certainty evidence. Fifth, sensitivity analyses and publication bias assessments supported the robustness of the findings. Finally, the biomarker analysis was strengthened by restricting quantitative synthesis to studies reporting BNP alone, thereby improving methodological consistency and reducing variability associated with pooling BNP and NT‐proBNP.

However, several limitations should be acknowledged. The inclusion of observational studies introduces the potential for residual confounding. Furthermore, one large observational cohort contributed substantially to the overall sample size and may have disproportionately influenced the pooled estimates for certain outcomes despite the additional subgroup and sensitivity analyses according to study design. Therefore, findings incorporating observational evidence should be interpreted alongside the randomized evidence. Second, important clinical heterogeneity existed across studies, including differences in ablation strategies, procedural technologies, atrial fibrillation subtype, baseline ventricular function, follow‐up duration, and background heart failure therapy, all of which may have influenced treatment effects. These factors could not be explored through detailed subgroup analyses because they were inconsistently reported across the included studies. Additionally, relatively few studies specifically enrolled patients with HFmrEF, and most evidence was derived from populations with predominantly reduced ejection fraction. Therefore, the applicability of these findings to patients with HFmrEF should be interpreted with caution. Furthermore, although LVEF and BNP were pooled using mean differences because they were reported on consistent measurement scales across studies, differences in imaging modalities for LVEF assessment and variability in BNP assay methods may have introduced measurement heterogeneity that could not be fully accounted for. Furthermore, LVEF and BNP were secondary or exploratory endpoints in most included studies rather than primary efficacy outcomes. Therefore, despite quantitative pooling, the corresponding estimates should be interpreted with caution. Certain outcomes, particularly BNP, were reported in a limited number of studies. In addition, differences in outcome definitions, especially for hospitalization, may limit comparability across studies.

## 6 Conclusion

In patients with atrial fibrillation and heart failure with reduced or mildly reduced ejection fraction, catheter ablation was associated with significant reductions in all‐cause and cardiovascular mortality, along with improvements in cardiac function and neurohormonal status. However, because most included studies predominantly enrolled patients with HFrEF, additional studies specifically involving HFmrEF populations are needed to confirm the generalizability of these findings.

## Author Contributions

Aleena Amir Malik and Kashif Yousaf conceptualized and supervised the study, contributed to the interpretation of findings, and drafted and critically revised the manuscript. Nasir Saeed, Goodluck Oparaugo, and Muhammad Waqar Shahid contributed to study design, interpretation of data, and drafting of the discussion section. Waqas Kareem, Fatima Yousaf, and Zain Ul Abedeen contributed to data interpretation and preparation of the results section. Fatima Sajjad managed PROSPERO registration, prepared the baseline characteristics tables, and assisted in methodological review. Fizza Kazmi drafted the methodology section and assisted with literature organization. Rubab Zahra prepared the introduction section and contributed to manuscript editing. Ishfaq Ali developed the PRISMA flow diagram and assisted with study selection documentation. Sahil Kumar and Tanzeela Gul performed risk of bias assessment and GRADE certainty evaluation of the included studies. Zartasha Rahim resolved data extraction discrepancies and assisted with data validation. Abdul Basit and Manal Sharif conducted data extraction and database organization. Anum Noor resolved disagreements during screening and eligibility assessment and contributed to critical manuscript revision. Habib ur Rehman and Imdad Ullah Shah performed literature screening and full‐text eligibility assessment. Abdullah Afridi conducted statistical analysis, interpreted pooled outcomes, and provided methodological oversight for the statistical and GRADE analyses. Goodluck Oparaugo and Waqas Kareem contributed to the preparation of the revised manuscript, preparation of the forest plots and graphical abstract, interpretation of data, and critical revision of the manuscript for important intellectual content. All authors critically revised the manuscript, approved the final version, and agreed to be accountable for all aspects of the work.

## Funding

The authors have nothing to report.

## Disclosure

Permission to reproduce material: No copyrighted material from other sources has been reproduced in this manuscript. All figures and tables were created by the authors.

## Ethics Statement

The authors have nothing to report.

## Consent

The authors have nothing to report.

## Conflicts of Interest

The authors declare no conflicts of interest.

## Supporting information


**Table S1:** Definition of outcomes.
**Table S2:** Newcastle–Ottawa Scale for quality assessment of cohort studies.
**Table S3:** GRADE assessment.
**Figure S1:** Traffic plot of risk‐of‐bias assessment using ROB tool.
**Figure S2:** Summary plot of risk‐of‐bias assessment.
**Figure S3:** Forest plot of all‐cause mortality for subgroup analysis according to baseline LVEF.
**Figure S4:** Funnel plot of all‐cause mortality.
**Figure S5:** Forest plot of cardiovascular mortality for subgrouping according to baseline LVEF.
**Figure S6:** Funnel plot of change in left ventricular ejection fraction.
**Figure S7:** Forest plot of change in LVEF for subgrouping based on baseline LVEF.
**Figure S8:** Forest plot of cardiovascular hospitalization after sensitivity analysis.

## Data Availability

Data sharing not applicable to this article as no datasets were generated or analysed during the current study.
