# Supplementary material for: Catheter Ablation Versus Medical Therapy in Atrial Fibrillation With Heart Failure With Reduced or Mildly Reduced Ejection Fraction: A GRADE‐Assessed Systematic Review and Meta‐Analysis
Source: J Arrhythm. 2026 Sep 21;42(5):e70464. doi: 10.1002/joa3.70464 (PMC13591214; doi:10.1002/joa3.70464)
Supplement: Supplementary file 1 [file JOA3-42-e70464-s001.docx]

| **Primary Outcomes** | **Outcome** | **Definition** |
| --- | --- | --- |
| 1 | All-Cause Mortality | Death from any cause occurring during the follow-up period, regardless of whether it is related to cardiovascular disease or other conditions. |
| 2 | Cardiovascular Mortality | Death attributed to cardiovascular causes, including heart failure, myocardial infarction, stroke, fatal arrhythmias, sudden cardiac death, or other vascular causes. |
| 3 | Change in Left Ventricular Ejection Fraction (LVEF) | Difference between baseline and follow-up left ventricular ejection fraction, measured as a percentage using the imaging modality reported in the original study (predominantly echocardiography, with cardiac MRI where applicable). |
| **Secondary Outcomes** | **Outcome** | **Definitions** |
| 1 | Change in B-type Natriuretic Peptide (BNP) | Difference between baseline and follow-up plasma B-type natriuretic peptide (BNP) concentration (pg/mL), reflecting changes in cardiac wall stress and heart failure severity. Studies reporting NT-proBNP were not pooled in this analysis. |
| 2 | Cardiovascular Hospitalization | Hospital admission due to cardiovascular causes. Across the included studies, this generally included hospitalization for worsening heart failure, atrial fibrillation–related events, or other cardiovascular complications. Minor variations existed in the operational definitions across studies, and the original study-specific definitions were adopted for data extraction and pooling. |

**Supplementary Table S1:** Definition of Outcomes

| **STUDY ID** | **SELECTION** | | | | **COMPARIBILITY** | | **OUTCOMES** | | | **TOTAL** |
| --- | --- | --- | --- | --- | --- | --- | --- | --- | --- | --- |
|  | **S1** | **S2** | **S3** | **S4** | **C1** | **C2** | **O1** | **O2** | **O3** |  |
| Lee 2022 | * | * | * | * | * | * | * | * | * | 9 |
| Long 2019 | * | * | * | - | * | - | * | * | * | 7 |
| Nor 2025 | * | * | * | * | - | * | * | * | * | 7 |

**Supplementary Table S2:** New Castle Ottawa Scale for Quality Assessment of Cohort Studies

| **Outcome** | **No. of Studies** | **Sample Size (Events)** | **Effect Estimate (95% CI)** | **Risk of Bias** | **Inconsistency** | **Indirectness** | **Imprecision** | **Publication Bias** | **Overall Certainty** |
| --- | --- | --- | --- | --- | --- | --- | --- | --- | --- |
| **All-Cause Mortality** | 10 | 131,229 (1,500 vs 2,546 events) | RR 0.61 (0.51–0.71) | Not serious¹ | Not serious (I²=15%) | Not serious | Not serious | Undetected | **⊕⊕⊕⊕ High** |
| **Cardiovascular Mortality** | 6 | 1,127 (35 vs 83 events) | RR 0.46 (0.31–0.66) | Not serious¹ | Not serious (I²=0%) | Not serious | Not serious | Undetected | **⊕⊕⊕⊕ High** |
| **Change in LVEF** | 11 | 1,706 (865 vs 841 participants) | MD 6.47% (4.47–8.47) | Not serious¹ | Serious² (I²=86%) | Not serious | Not serious | Undetected | **⊕⊕⊕◯ Moderate** |
| **Change in BNP** | 3 | 190 (96 vs 94 participants) | MD −136.85 pg/mL (−190.46 to −83.23) | Not serious¹ | Not serious (I²=21%) | Not serious | Serious³ | Undetected | **⊕⊕⊕◯ Moderate** |
| **Cardiovascular Hospitalization** | 5 | 130,661 (41,383 vs 34,580 events) | RR 0.78 (0.53–1.13) | Not serious¹ | Serious² (I²=91%) | Not serious | Not serious | Undetected | **⊕⊕⊕◯ Moderate** |

**¹ Risk of bias:** Randomized controlled trials were assessed using the **Cochrane RoB 2 tool**, with most studies judged to have a low risk of bias and three studies having some concerns. Cohort studies were assessed using the **Newcastle–Ottawa Scale (NOS)**, with Lee et al. scoring 9/9 stars and Long et al. and Nor et al. scoring 7/9 stars, indicating overall moderate-to-high methodological quality.

**² Serious inconsistency:** Downgraded one level because of substantial heterogeneity (LVEF: I² = 86%; cardiovascular hospitalization: I² = 91%).

**³ Serious imprecision:** Downgraded one level because the evidence was derived from only three relatively small studies.

**Supplementary Table S3:** Grade Assessment

**Supplementary Figure S1:** Traffic plot of Risk of Bias Assessment using ROB tool

**Supplementary Figure S2:** Summary Plot of Risk of Bias Assessment

**Supplementary Figure S3:** Forest Plot of All Cause Mortality for subgroup analysis according to baseline LVEF

**Supplementary Figure S4:** Funnel Plot of All Cause Mortality

**Supplementary Figure S5:** Forest plot of Cardiovascular Mortality for subgrouping according to baseline LVEF

**Supplementary Figure S6:** Funnel Plot of Change in Left Ventricular Ejection Fraction

**Supplementary Figure S7:** Forest Plot of Change in LVEF for subgrouping based on baseline LVEF

**Supplementary Figure S8:** Forest Plot of Cardiovascular Hospitalization after Sensitivity Analysis
